# High-Strength Konjac Glucomannan/Silver Nanowires Composite Films with Antibacterial Properties

**DOI:** 10.3390/ma10050524

**Published:** 2017-05-12

**Authors:** Jia Lei, Lei Zhou, Yongjian Tang, Yong Luo, Tao Duan, Wenkun Zhu

**Affiliations:** 1 Sichuan Civil-Military Integration Institute, Mianyang 621010, China; cutiancheng@163.com (J.L.); 18281605998@163.com (L.Z.); yjtang@swust.edu.cn (Y.T.); 2 Sichuan Biotech Force Technology Co., Ltd., Mianyang 621010, China; kaientelo@gmail.com; 3 School of National Defense Science and Technology, Southwest University of Science and Technology, Mianyang 621010, China

**Keywords:** konjac glucomannan, silver nanowires, film, high strength, antimicrobial activity

## Abstract

Robust, high-strength and environmentally friendly antibacterial composite films were prepared by simply blending konjac glucomannan (KGM) and silver nanowires (Ag NWs) in an aqueous system. The samples were then characterized using scanning electron microscopy (SEM), X-ray diffraction (XRD), thermal gravimetric analysis, mechanical property tests, Fourier transform infrared spectra (FT-IR), X-ray photoelectron spectroscopy (XPS) and antimicrobial tests. The results showed that there was a high ratio of Ag NWs uniformly distributed in the composite films, which was vital for mechanical reinforcement and stable antibacterial properties. The enhanced thermal stability and mechanical intensity increased, while the elongation at break was reduced with an increase in the amount of Ag NWs found in the composite films. When the percentage of Ag NWs in the composite films reached 5%, the tensile strength was 148.21 MPa, Young’s modulus was 13.79 GPa and the ultimate strain was 25.28%. Antibacterial tests showed that the KGM films had no antibacterial effect. After the addition of Ag NWs, the composite films had an obvious inhibitory effect on bacteria, with the uniform dispersion of Ag NWs promoting the antibacterial effect to a certain degree. These results indicated that these composite films would have a potential application in the fields of environmentally friendly packaging or medicine.

## 1. Introduction

At present, most composite films are made of petroleum chemical polymers [1], which places an enormous amount of pressure on energy resources and the environment. It is reasonable to use natural polymers instead of petroleum products to save the non-renewable fossil fuels. In past decades, considerable attention has been given to investigating the new applications of natural polymers. A number of natural polymers and their derivatives were widely used as new functional materials in biochemical, industrial, medical and controlled-release fields. Konjac glucomannan (KGM), consisting of β-l,4 linked D-mannose and D-glucose, is a naturally renewable polysaccharide polymer from the tuber of the Amorphophallus konjac plant [2]. Generally, KGM has biodegradability [3], hydrophilicity [4], gel properties [5], film-forming ability [6,7], low caloric value, some special physiological functions and qualifiable functionalization [8]. KGM and KGM derivatives could be widely used in food, medicine, chemical and biological fields [9].

Although KGM has a variety of properties, pure KGM film has only a single function. To expand the applications of KGM films, nanotechnology has provided a new way for the preparation of composite materials with superior properties that may exceed the individual components [10]. Inorganic nanomaterial was introduced into KGM for organic/inorganic nanocomposites, as the small size effect of nanomaterials and the interaction between the matrices of the composite materials would improve the strength, stiffness, toughness and certain functionalities of the composite material [11,12]. However, the inorganic components in the polymer/inorganic nanocomposite films are mostly nanoparticles [13,14]. These nanoparticles can endow the material with their corresponding functional properties [15], which has little effect on the mechanical properties of the composite films. For one-dimensional metallic nanomaterials, especially for nanowires, the network structure can be formed in the polymer, which can improve the mechanical properties and provide the functional properties of the original material [16,17].

One-dimensional nanomaterials, such as silver nanowires (Ag NWs), prepared by template and template-free methods, have attracted great attention from electronic and chemistry researchers for their unique and controllable chemical, plasmonics, optical, beneficial catalytic properties and other related properties [18,19]. In addition, they can be used in electronics [20], catalysis, medicine, energy, information storage areas and so on. Recently, focus has been placed on fabricating Ag NW-related composites, due to their unique structure and nature [21,22], mechanical properties, antibacterial properties [23,24] and the biocompatibility of Ag NWs potentially being transferred to the composite materials. Some polymers, such as polyvinyl alcohol, chitosan and resins, have been used as substrates for the Ag NWs to achieve high-strength antibacterial properties and chemical stability. However, there have been no reports of Ag NWs being added to the natural polymer konjac glucomannan for composite films.

In this present study, we fabricated a robust and antibacterial composite film by simply blending KGM and Ag NWs in an aqueous system, with Ag NWs being uniformly distributed in the composite film. Experiments on the syntheses, morphology, composition and structure, thermal stability, mechanical properties, rheological property and antibacterial property were also carried out. This composite film showed good thermostability, mechanical performance and antibacterial properties due to the unique character of the nanocomposites, which may show a promising application in the fields of environmentally friendly packaging and medicine.

## 2. Materials and Methods

### 2.1. Materials 

Purified KGM was purchased from the Enshi Hongye Konjac Development Co., Ltd. (Enshi, China). Silver nitrate (AgNO_3_) was purchased from the PLA 9509 factory (Wuhan, China). Polyvinyl-pyrrolidone (PVP) was purchased from the Regent Tianjin Chemical Co., Ltd. (Tianjin, China). Ethylene glycol ((CH_2_OH)_2_) was purchased from the Tianjin Fu Yu Fine Chemical Co., Ltd. (Tianjin, China). Acetone (CH_3_COCH_3_) was purchased from the Nanjing Chemical Reagent Co., Ltd. (Nanjing, China). Absolute ethyl alcohol (C₂H₆O) was purchased from the Tianjin Chemical Reagent Factory (Tianjin, China). Tris (NH_2_C(CH_2_OH)_3_) was purchased from the Sinopharm Chemical Reagent Factory (Shanghai, China). Hydrochloric acid (HCl) was purchased from the Chengdu Kelong Chemical Reagent Factory (Chengdu, China).

### 2.2. Synthesis of Ag NWs 

Ag NWs were prepared using a solvothermal method [25]. A total of 10 mL of the PVP-(CH_2_OH)_2_ solution (0.5 mol/L) was added to a 50-mL three-necked flask, before being uniformly mixed and heated in an oil bath at 150 °C for 1.5 h. A total of 10 mL of the AgNO_3_-(CH_2_OH)_2_ solution (0.2 mol/L) was added to the three-necked flask, with heating stopped after 1 h of reaction time. Throughout the reaction, the rotor speed was 1200 rpm. The color of the reacting solution gradually changed from bright yellow to orange, before finally becoming pale. CH_3_COCH_3_ was added into the reaction solution twice, before undergoing ultrasonic washes. Following this, the resulting product was placed in the centrifuge at 5000 rpm for 30 min. After the upper section of the solution was discarded, the precipitate was dispersed in 95% anhydrous ethanol to obtain the final Ag NWs sample.

### 2.3. Synthesis of Composite Film

**Figure 1:**
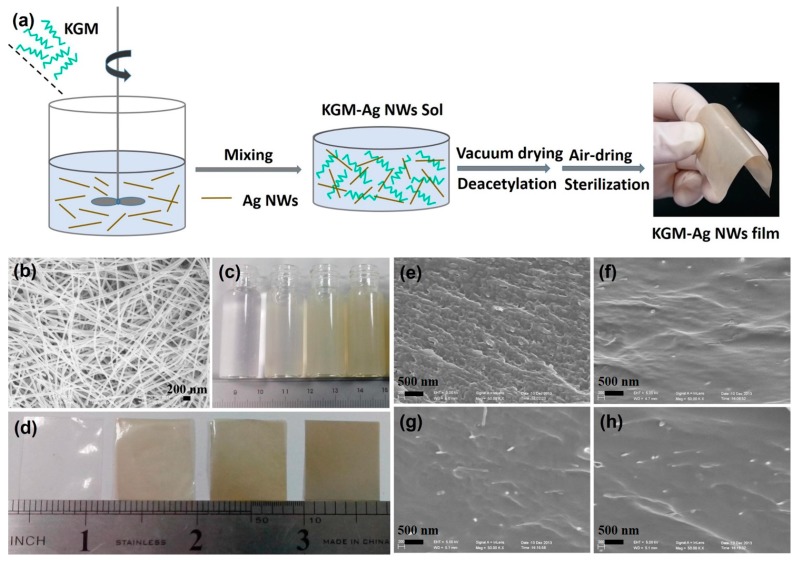
(**a**) Schematic representation for synthesis of hybrid film; (**b**) scanning electron microscopy (SEM) images of Ag NWs; photos of the KGM and composite (**c**) solutions; (**d**) films with increasing % wt of Ag NWs; cross-sectional SEM images of (**e**) KGM film; (**f**) KGM-Ag NWs-1%; (**g**) KGM-Ag NWs-2.5%; and (**h**) KGM-Ag NWs-5% composite films.

The 0%, 1%, 2.5% and 5% (w/w) Ag NWs were dispersed in 50 mL of distilled water and 1% (w/w) KGM was dissolved in the solution of Ag NWs by constantly stirring for 0.5 h in a water bath at 60 °C. Next, the mixed solution was poured into a Petri dish before being vacuum-dried at 60 °C for 12 h. The dried composite films were immersed in a 2% (v/v) sodium hydroxide ethanol solution at 65 °C for 5 h. Following this, the films were removed, washed with water until neutral and then air-dried in a sterilization station at 60 °C for 12 h to obtain the dry composite films. The dried films were placed into a desiccator with 57% relative humidity (saturated solution of sodium bromide) for use. The schematic diagram of the production process of the composite film is shown in Figure 1a.

### 2.4. Characterization

Film thickness was measured using a Vernier caliper with a standard error of ±0.01 from five different points, with the mean used in the calculation. Water content was calculated by weighing the films before and after drying at 60 °C. Transparency was measured by UV spectrophotometry (UV-2450, SHIMADZU, Tokyo, Japan). Water vapor permeability (WVP) tests were conducted following the ASTM (1981a) Method E96-80, with some modifications [26]. Scanning electron microscopy (JSM-6390/LV, Jeol, Tokyo, Japan) was utilized to characterize the structure of Ag NWs and the cross-sections of films, with the samples then being fractured in liquid nitrogen. X-ray diffraction (X Pert pro, PANalytical, Almelo, The Netherlands) patterns of the films were carried out with CuK(α) radiation at a voltage of 60 kV and a current of 50 mA. Thermal gravimetric analyses (TGA) was carried out using a thermal gravimetric analysis instrument (Q5000, TA, Newark, NJ, USA) under nitrogen at a heating rate of 10 °C/min. The mechanical properties of films were measured by a static mechanical tester (Instron 5565A, INSTRON, Shanghai, China) under the tensile mode. During the measurement of the mechanical properties, the distance between the two fixtures equaled 5 mm, the moving speed was 10 mm/min and the films were cut into 23 mm × 5 mm (length × width) rectangles before testing. X-ray photoelectron spectroscopy (XPS) analyses were carried out using a Kratos Axis Ultra (Kratos Analytical, Kratos Tech, Manchester, UK) photoelectron spectrometer. This instrument uses a monochromatic Al K_α_ X-ray source. Fourier transform infrared spectroscopy (FT-IR) spectra of films were taken by a Nicolet Avatar 370 FT-IR spectrometer (Thermo Nicolet, Waltham, MA, USA) at wavelength range of 4000–400 cm^−1^. Rheological analysis was measured by a HAAKE RS6000 Rotational Rheometer (Thermo Hakke, Hamburg, Germany). For this analysis, the frequency sweep was conducted from 0.01 to 100 rad/s, with the 1-mm plate geometry rotor at 25 ± 0.05 °C and a deformation of 0.5%, which was within the linear viscoelastic range (LVE). 

### 2.5. Antimicrobial Tests

The sterilized agar nutrient solution was melted and kept at 50 °C for 0.5 h. Next, the solution was poured onto a sterile disposable petri dish, before being cooled and solidified. A total of 100 μL of the overnight culture of *E. coli* and *S. aureus* (10^6^ CFU/mL) was spread evenly across the plates, before the composite films and the KGM film were carefully placed in the medium containing *E. coli*. After the overnight incubation at 37 °C, the size of the inhibition zone was observed. 

## 3. Results and Discussion

### 3.1. Basic Information

We controlled for the thickness of the KGM film and all KGM/Ag NWs films, which were all found to have relatively rough film surfaces. The film thickness and water content also did not show any significant differences between the KGM film and KGM/Ag NWs films. As shown in Table 1, the transparency of the KGM film was 85%, with the transparency of films decreasing sharply with an increase in the concentration of Ag NWs. When the concentration of Ag NWs was 5%, the transparency was 3%, making it almost impossible to pass light through these films. The water vapor permeability rate of the film showed a similar trend. The water vapor permeability of the KGM film was 1.36 × 10^14^ Kg Pa^−1^ s^−1^ m^−1^, which was reduced to 0.3 × 10^14^ Kg Pa^−1^ s^−1^ m^−1^ when the concentration of Ag NWs was 5%. This was due to the production of a large number of Ag NWs after the composite films were created. Figure 1b depicts the typical SEM images of Ag NWs, demonstrating the successful fabrication of Ag NWs by the chemical reduction method. The Ag NWs had a uniform linear shape with a high aspect ratio of about 1300. The diameter of the Ag NWs was about 30–40 nm and the length was more than 50 μm. Figure 1c,d show KGM and KGM/Ag NWs composite solutions and films, respectively. The images show that the KGM film was colorless and transparent. As the amount of Ag NWs increased, the color of the solution and films gradually increased, eventually becoming opaque. In addition, the composite KGM/Ag NWs films exhibited flexibility and could be bent naturally. Figure 1e shows the cross-sectional image of the KGM film, while Figure 1f–h are cross-sectional SEM images of the composite films with different concentrations of Ag NWs. The cross-sectional surface of the KGM film was rough, while buried silver nanowires were obvious in the composite films with an increase in their concentration. The cross-sectional surface of the composite films was smooth, because the addition of Ag NWs made the films more compact.

### 3.2. X-ray Diffraction (XRD) and Thermal Gravimetric Analysis

**Figure 2:**
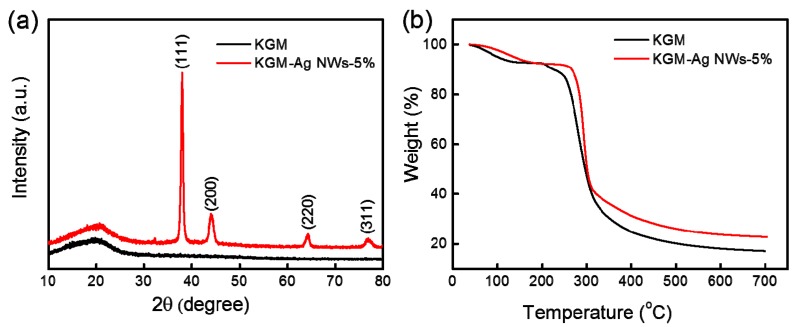
(**a**) X-ray diffraction (XRD) patterns for the KGM film and the KGM-Ag NWs-5% composite film; (**b**) Thermal gravimetric analyses (TGA) results of the KGM film and the KGM-Ag NWs-5% composite film.

Figure 2a shows the XRD patterns of the KGM-Ag NWs-5% composite film, which uses the KGM film as the control sample. The XRD spectra of the KGM film had no obvious diffraction peaks, due to the low crystallinity. As for the KGM-Ag NWs-5% composite film, four peaks at 2θ = 38.1°, 44.4°, 64.4° and 77.5° are presented, which correspond sequentially to the (111), (200), (220) and (311) of silver, which is consistent with the Joint Committee On Powder Diffraction Standards (JCPDS) file (No. 89-3722) [27].

Figure 2b shows the TGA data of the KGM film and the KGM-Ag NWs-5% composite film. As shown in Figure 2b, the thermal decomposition of the KGM film was divided into two stages [28]. The first stage ranges from 38 to 175 °C, corresponding with a weight loss of 7.25%. This weight loss was due to the loss of free water and part of the combined water, due to the absorption of water in the –OH and non-crystalline regions. The second stage was from 175 to 354 °C. This was the process of degradation and cracking of the polymer chain. The rate of weight loss was the fastest in this process, with the weight loss being 74.21%. From 354 to 700 °C, the remaining residue of KGM was carbon-black in color and its corresponding content was about 17.21%. When Ag NWs were added, the decomposition of the composite film in the first stage was significantly delayed at 208 °C. The rate of weight loss slowed down, but the weight loss did not change significantly. In the second stage without adding silver nanowires, weight loss decreased to 11.62%. Finally, the residue was 22.84%. The data showed that the thermal stability of the composite film was increased by adding Ag NWs. This was probably due to the Ag NWs forming a reticular structure between KGM molecules and acting as the crosslinking point in the composite films, which control thermal motions of the polymer matrix in the composite film [29].

### 3.3. Mechanical Performance

**Figure 3:**
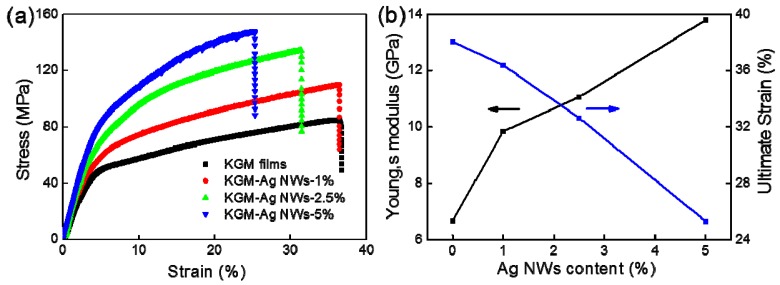
(**a**) The stress-strain curves of the KGM film and the KGM/Ag NWs films; (**b**) the dependence of Young’s modulus and the ultimate strain on the content of Ag NWs for composite films.

The mechanical properties of the composite film were enhanced with the addition of the Ag NWs. The stress-strain curves of the KGM film and the KGM/Ag NWs films are shown in Figure 3a. The tensile strength of the pure KGM film was 81.9 MPa and the strain was 37.4%. When the content of the Ag NWs was 1%, the tensile strength increased by 40%. However, the strain of the composite film did not change. With an increase in the tensile strength, the strain on the composite film became smaller. When the content of the Ag NWs was 5%, the tensile strength increased by 85% to reach a maximum of 148.21 MPa, while the strain decreased by 32%. This showed that a small amount of silver nanowires (~1%) had no effect on the flexibility of the film. However, continuously adding silver nanowires could reduce the flexibility of the composite film.

The dependence of Young’s modulus and the ultimate strain on the content of Ag NWs for composite films are shown in Figure 3b. Young’s modulus of the KGM film was 6.67 GPa, and the ultimate strain was 38.05%. Young’s modulus of the KGM/Ag NWs film with 5% of Ag NWs was 13.79 GPa and the ultimate strain was 25.28%. Results show that Young’s modulus of the composite film increased with the addition of Ag NWs, but the ultimate strain was reduced. Ag NWs in the KGM/Ag NWs films can improve the mechanical properties in a similar way to how other flakes or inorganic fibers improve the mechanical properties in reinforced polymers [30]. It is well-understood that Ag NWs prepared by the polyol process have a high tensile strength [31], which can reinforce the KGM/Ag NWs films when breaking occurs. Ag NWs played a significant reinforcement effect, which restricted the growth of the KGM matrix and Ag NWs in addition to resulting in beneficial tensile properties. It is apparent that the Ag NWs forming network structures can suppress crack propagation in the KGM films and generate a more uniform strength.

### 3.4. FT-IR Analysis

**Figure 4:**
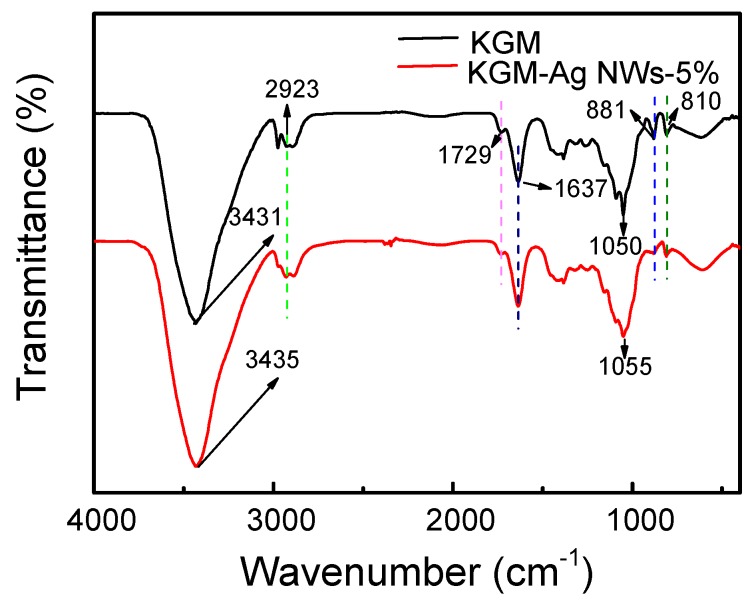
Fourier transform infrared spectroscopy (FT-IR) spectrum of the KGM and KGM-Ag NWs-5% films.

The FT-IR spectra of the KGM and KGM-Ag NWs-5% films with a wavelength range of 4000–400 cm^−1^ is shown in Figure 4. The absorption bands at 3431 cm^−1^ and the peaks at 2923 cm^−1^ of KGM were assigned to the stretching of –OH groups and C–H of methyl in KGM. The absorption bands at 1729, 1637 and 1050 cm^−1^ were assigned to the stretching of C=O, C–O, and C–O–H groups. The absorption bands at 881 and 810 cm^−1^ were characteristic vibrations of the mannose unit in KGM. By comparing the spectra of KGM and KGM-Ag NWs-5% film, the –OH and C–O–H stretching peaks moved to the high wavelengths of 3435 cm^−1^ and 1055 cm^−1^, respectively, which was due to the damage of hydrogen bonding from the hydration between the KGM molecular chain and the water molecule in alkaline solutions [32,33]. Thus, this implies an interaction between the –OH groups of KGM and the Ag NWs in the KGM-Ag NWs-5% films [34]. The existence of these interactions in the assembly films would further explain the resultant mechanical reinforcement effect.

### 3.5. Rheological Analysis

**Figure 5:**
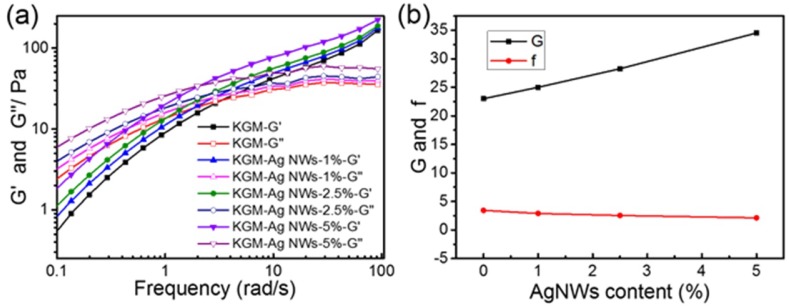
(**a**) The frequency sweep of KGM solution and KGM/Ag NWs solution; (**b**) statistics on the intersection modulus and crossover frequency in different amounts of Ag NWs.

Figure 5a depicts the changes in storage (G′) and loss modulus (G″) in the frequency sweep of the KGM solution and KGM/Ag NWs solution. Under the same concentration of KGM, G′ and G″ all increased gradually with the increase of Ag NWs, which illustrated that the interaction between Ag NWs and KGM has been enhanced. Ag NWs between the KGM polyhydroxy substances formed complex bridges, which increased the interaction forces between molecular chains. Figure 5b depicts the statistics regarding the intersection modulus and crossover frequency in different amounts of Ag NWs. With an increasing concentration of Ag NWs, the intersection modulus of G was increased up to a maximum of 50% higher. However, the crossover frequency was decreased by about 40%. This means that it had higher mechanical properties, which could resist at a lower frequency and for a longer time. This was due to the polyhydroxy of KGM and Ag NWs. The density of molecular chains was obviously improved, with an increase in the crosslinking point and entanglement of molecular chains.

### 3.6. XPS Analysis

**Figure 6:**
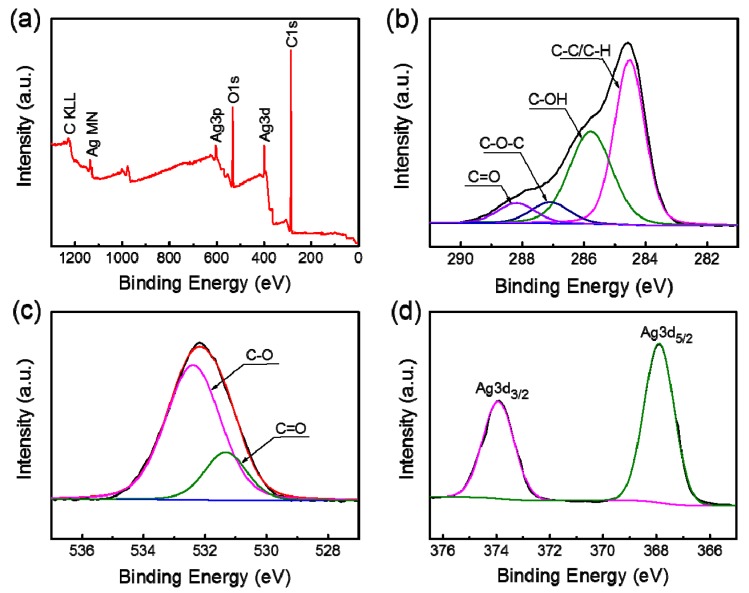
(**a**) XPS spectrum of the KGM-Ag NWs-5% composite film; high-resolution spectra of samples for the elements of (**b**) C, (**c**) O and (**d**) Ag.

XPS was used to analyze the chemical functionalities and covalent bonds in the KGM/Ag NWs film. The survey spectrum showed the presence of C, O and Ag elements (Figure 6a). The high resolution of the C1s XPS spectra was shown in Figure 6b. There were four fitting peaks in the C1s curve, including C–C/C–H (284.5 eV), C–OH (285.8 eV), C–O–C (287.1 eV) and C=O (288.2 eV). The O1s peak (Figure 6c) fit into two components and the peaks indicated two different types of oxygen linkages. The lower binding energy (531.3 eV) stands for oxygen (labeled as O1) bonded to carbon in a double bond (C=O), while the higher one (labeled as O2) (532.4 eV) was produced by the C–O bond. As shown in Figure 6d, the appearance of two peaks centered at 367.9 eV and 373.9 eV were assigned to Ag 3d_5/2_ and Ag 3d_3/2_, respectively, revealing that the Ag NWs were in the metallic state in the KGM/Ag NWs composite film.

### 3.7. Antimicrobial Tests

**Figure 7:**
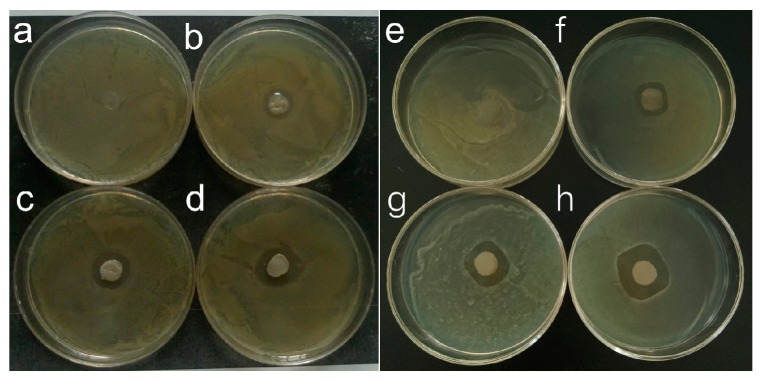
Photos of inhibitory zones for *E. coli* using the: (**a**) KGM film, (**b**) KGM-Ag NWs-1% film, (**c**) KGM-Ag NWs-2.5% film and (**d**) KGM-Ag NWs-5% film; and for *S. aureus* using the: (**e**) KGM film, (**f**) KGM-Ag NWs-1% film, (**g**) KGM-Ag NWs-2.5% film and (**h**) KGM-Ag NWs-5% film.

It is common knowledge that nanoparticles of silver have excellent antimicrobial activity. However, in previous studies, the antimicrobial activity of Ag NWs was rarely reported. For example, Shahzadi et al. [30] reported a type of CS/Ag NWs film had a good antibacterial effect on *E. coli* and *S. aureus*. In this antifungal test, we found that there was no inhibition zone in the KGM antibacterial test (Figure 7a,e), which was consistent with the antimicrobial activity found for the pure KGM film in previous research [35]. However, the KGM solution has antimicrobial activity, while pure KGM films are not antimicrobial. In addition, the inhibition zone of *E. coli* and *S. aureus* gradually increased with increasing amounts of Ag NWs. The calculated average inhibition zones of *E. coli* were approximately 8.4, 11.2 and 14.6 mm (Figure 7b–d), while these average zones were 9, 13.6 and 16 mm for *S. aureus* (Figure 7f–h). The nano-size and the large specific surface area of Ag NWs was beneficial for the adsorption of the bacterial cell membrane and combined with the enzyme thiol (–SH) involved in metabolism to inactivate this enzyme, resulting in a blockage of metabolism [36]. The dissolution of Ag NWs allowed these particles to enter the bacteria and produce an inhibitory effect. In addition, the uniform distribution of Ag NWs in the KGM film also promoted the antibacterial property of the composite film to a certain degree.

## 4. Conclusions

We fabricated robust and antibacterial composite films of KGM and Ag NWs, with Ag NWs uniformly distributed in the composite films. After the addition of Ag NWs, the tensile strength of the composite films was increased, while the strain was decreased. The weight loss of the composite film was lowered and the thermal stability of the composite film was improved. When the content of Ag NWs reached 5%, the tensile strength was 148.21 MPa and Young’s modulus was 13.79 GPa. The KGM film had no antibacterial effect. After the addition of Ag NWs, the composite films had an obvious bacterial inhibitory effect and the uniform dispersion of Ag NWs promoted the antibacterial effect to a certain degree. We predict that this high-strength KGM/Ag NWs composite film would have potential applications as environmentally friendly packaging or in medicine in the future.

## Figures and Tables

**Table 1:** Thickness, transparency, water vapor permeability and water content of Konjac glucomannan (KGM) and hybrid films with different concentrations.

Samples	Thickness (mm)	Transparency (%)	WVP × 10^14^ (Kg Pa^−1^ s^−1^ m^−1^)	Water Content (%)
KGM	0.16 ± 0.01	85 ± 0.8	1.36 ± 0.19	0.21 ± 0.06
KGM-Ag NWs-1%	0.17 ± 0.02	43 ± 0.5	1.16 ± 0.25	0.19 ± 0.07
KGM-Ag NWs-2.5%	0.19 ± 0.02	20 ± 0.6	0.8 ± 0.17	0.18 ± 0.05
KGM-Ag NWs-5%	0.20 ± 0.03	3 ± 0.2	0.3 ± 0.04	0.16 ± 0.03
